# Suppressing Unwanted Memories Reduces Their Unintended Influences

**DOI:** 10.1177/0963721417689881

**Published:** 2017-04-06

**Authors:** Xiaoqing Hu, Zara M. Bergström, Pierre Gagnepain, Michael C. Anderson

**Affiliations:** 1 Department of Psychology, The University of Hong Kong; 2 The State Key Laboratory of Brain and Cognitive Science, The University of Hong Kong; 3 School of Psychology, University of Kent; 4 Normandie Université, UNICAEN, PSL Research University, EPHE, INSERM U1077, CHU de Caen, Neuropsychologie et Imagerie de la Mémoire Humaine, Caen, France; 5 Medical Research Council Cognition and Brain Sciences Unit, University of Cambridge; 6 Behavioral and Clinical Neurosciences Institute, University of Cambridge

**Keywords:** retrieval suppression, explicit/implicit memory, suppression-induced forgetting, direct/indirect memory tests

## Abstract

The ability to control unwanted memories is critical for maintaining cognitive function and mental health. Prior research has shown that suppressing the retrieval of unwanted memories impairs their retention, as measured using intentional (direct) memory tests. Here, we review emerging evidence revealing that retrieval suppression can also reduce the unintended influence of suppressed traces. In particular, retrieval suppression (a) gradually diminishes the tendency for memories to intrude into awareness and (b) reduces memories’ unintended expressions on indirect memory tests. We present a neural account in which, during suppression, retrieval cues elicit hippocampally triggered neocortical activity that briefly reinstates features of the original event, which, in turn, are suppressed by targeted neocortical and hippocampal inhibition. This reactivation-dependent reinstatement principle could provide a broad mechanism by which suppressing retrieval of intrusive memories limits their indirect influences.

Blessed are the forgetful; for they get the better even of their blunders.

—Friedrich Nietzsche, *Beyond Good and Evil: Prelude to a Philosophy of the Future*

Not all memories are equally welcome. Contrary to the commonly held belief that forgetting is undesired and to be circumvented, there are many everyday situations in which we would rather not recall certain memories. For example, confronting a reminder of a previous relationship can call to mind intrusive memories that occupy our consciousness, causing distress and distraction. Understandably, people often avoid such reminders as a way of managing thoughts about an unpleasant past. Reminders can, however, be unavoidable. People, places, or objects may resemble, perceptually or conceptually, features of unwanted memories and trigger unwelcome retrievals; when this happens, people often suppress the retrieval process to stop the unwanted memories from coming to mind, which may reduce their later accessibility.

Retrieval suppression has been studied extensively using the think/no-think (TNT) paradigm (Anderson & Green, 2001; for a recent review, see Anderson & Hanslmayr, 2014). In this procedure (Fig. 1a), people learn cue-target pairs and are then given the cues again with instructions either to retrieve (i.e., “think”) or to stop retrieval of (i.e., “no-think”) the associated target memories while also sustaining attention to the cue. Critically, performing the latter no-think task requires that people override the cue’s strong tendency to elicit automatic retrieval of its associated memory. Behavioral and neuroimaging evidence suggests that such retrieval suppression engages inhibitory control mechanisms that enable people to stop habitual response tendencies, such as reflexive motor responses or thoughts (see Anderson et al., 2004; Depue, Orr, Smolker, Naaz, & Banich, 2016). Evidence of inhibition can be detected via suppression’s negative aftereffects on suppressed items: On episodic-memory tests, suppressed items are recalled more poorly than are baseline items, a phenomenon known as *suppression-induced forgetting*. The amount of forgetting increases with the number of times a memory has been suppressed, indicating that unwanted memories are cumulatively inhibited over repeated suppressions. A number of variables moderate the size and indeed the occurrence of this effect in explicit memory (e.g., compliance, vigilance; see Anderson & Huddleston, 2012, for a thorough review of key moderators). Retrieval-suppression research thus indicates that people can stop episodic retrieval and that this process causes forgetting on direct memory tests.

**Fig. 1.**
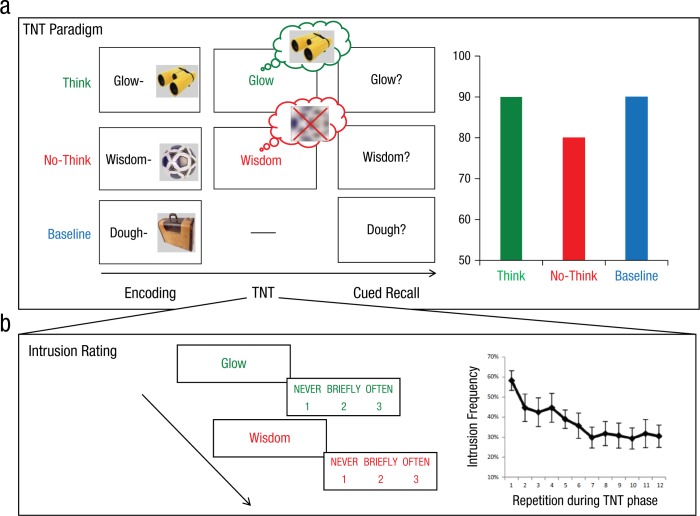
Procedural overview and results for a think/no-think task (TNT; a) and assessment of involuntary TNT intrusions (b). In the TNT paradigm (a), participants first learn cue-target pairs during the encoding session. During the TNT session, participants are repeatedly presented with the original cue words in either green (“think”) or red (“no-think”) font colors and are asked to think or to not think about the associated target memories, respectively. In a subsequent cued-recall session, participants are prompted to recall each target that was paired with the original cue word. Results show that repeatedly suppressing the no-think items (approximately 10–16 times) reduces the likelihood these memories can be recalled (Anderson & Green, 2001). This basic paradigm has been extended to investigate the suppression of different types of materials, and the consequences of suppression have been assessed with a variety of tests. On each trial of one study assessing involuntary intrusions during TNT sessions (Levy & Anderson, 2012), participants were asked to report how often they thought of the associated targets upon seeing think and no-think reminders (b). Involuntary intrusions on no-think trials, indicated by ratings of 2 or 3, declined with repeated suppression.

Until recently, however, it was unknown whether suppressing retrieval affects less conscious, unintentional retrieval of unwanted memories and, if so, how this might be achieved. We use *unintentional memory* throughout to refer to indirect expressions of memory as revealed by conventional tests of implicit memory as well as retrieval (conscious or not) that is elicited involuntarily upon encountering reminders, despite a lack of any intention to retrieve a memory. Here, we review emerging evidence indicating that retrieval suppression can diminish these unintentional expressions of memory, and we discuss the neural mechanisms underlying these effects.

## Why Study Unintentional Retrieval?

Explicit and implicit memories have often been dissociated (Schacter, 1987). Given this dissociation, retrieval suppression could, in principle, impair explicit retrieval while preserving unintended expressions of memory, allowing traces to exert potentially unwanted effects outside of awareness. A selective disruption of explicit memory would be compatible with evidence that retrieval suppression down-regulates activity in the hippocampus, a structure critical to the formation of episodic memories (Anderson et al., 2004; see Anderson & Hanslmayr, 2014, for a review), as well as event-related potential (ERP) activity associated with conscious recollection (Bergström, Velmans, De Fockert, & Richardson-Klavehn, 2007). Alternatively, if suppression also disrupts unintentional retrieval, this raises the possibility that cognitive or neurobiological theories of this process couched exclusively in terms of episodic memory do not capture key dynamics of the suppression mechanism and its targets.

Whether suppression reduces unintended retrieval also has implications for how it might affect mental health. In everyday life, people rarely intentionally recall unwanted memories, especially after they have tried to suppress them. Rather, the more practical concern is the tendency of unwanted memories either to intrude into awareness involuntarily or to influence behavior indirectly, in potentially unhealthy ways. Indeed, excessive intrusions arise in a range of psychopathologies including anxiety disorder, posttraumatic stress disorder (PTSD; Brewin, 2014), obsessive-compulsive disorder (Speckens, Hackmann, Ehlers, & Cuthber, 2007), and depression (Brewin, Gregory, Lipton, & Burgess, 2010) and often occur along with pathological rumination (Disner, Beevers, Haigh, & Beck, 2011). Intrusions are usually perceived as vivid, detailed, unexpected, uninvited, and uncontrollable. To resist intrusions, people may engage in self-distraction or avoidance of triggers, strategies that paradoxically are associated with increased thought frequency, hypervigilance, and negative appraisal of the meaning of intrusions (e.g., Purdon, 2004). For these reasons, some have argued that attempts to suppress intrusions are unhelpful and maladaptive (cf. Dunn, Billotti, Murphy, & Dalgleish, 2009). Some theoretical accounts even maintain that successfully forgotten memories continue to influence behavior and thought implicitly, undermining mental health (e.g., Berlin, 2011; Pennebaker & Susman, 1988; Schwartz, 1990). Although clinical observations about unconscious influences are widely discussed, research has not adequately separated the effects of avoidance (e.g., avoiding triggers) from those of retrieval suppression, which are theoretically distinct (Catarino, Küpper, Werner-Seidler, Dalgleish, & Anderson, 2015). As a result, without direct evidence concerning whether and how retrieval suppression influences unintended retrieval, one cannot evaluate its implications for mental health. Therefore, studying whether suppression affects unintentional retrieval may expand our understanding of this process and provide critical information about its clinical implications.

## Suppression Reduces Unintentional Memory Intrusions

How effective is retrieval suppression at mitigating the occurrence of automatic, intrusive retrievals? Does the fact that intrusive memories come to mind despite our intention to stop them mean that suppression is unlikely to be effective at countering them in the long run? One difficulty in studying this issue is in measuring involuntary retrievals in the laboratory. To answer these questions, Levy and Anderson (2012) conducted an experiment with the TNT task and asked participants to report on a trial-by-trial basis whether unwanted memories had intruded into awareness on the preceding no-think trial (Fig. 1b). Critically, because participants were striving to prevent the cue from eliciting retrieval of its associated memory on no-think trials, any retrieval that arose would be not only unintentional but also counter-intentional, happening despite efforts to stop it. Thus, intrusions during no-think trials provided a clear operational definition of involuntary memory. Levy and Anderson found that people did experience counter-intentional intrusions during retrieval suppression (for 60% of the items, on average, on the first suppression trial). However, participants dramatically decreased these intrusions across repeated suppressions (Fig. 1b; see also Benoit, Hulbert, Huddleston, & Anderson, 2015, and van Schie & Anderson, 2017). Interestingly, participants who reduced intrusions effectively also showed the greatest suppression-induced forgetting on the final test. This finding suggests that suppression reduces both unintentional retrievals during suppression attempts and later intentional retrieval, and that these effects are related. Reduced intrusions have been observed with pairs of words as well as with visual images (Benoit et al., 2015). The temporal dynamics of intrusions and their purging from working memory have, moreover, been documented with ERPs and linked to suppression-induced forgetting (Hellerstedt, Johansson, & Anderson, 2016).

Does the ability to suppress retrieval predict how well people regulate intrusive emotional memories? Recently, Streb, Mecklinger, Anderson, Johanna, and Michael (2016) examined this issue using the *trauma film paradigm* (Holmes & Bourne, 2008). Participants first completed the TNT task with simple word pairs, and both behavioral (suppression-induced forgetting) and ERP (the N2 component) measures of memory-control ability were computed. Next, participants viewed a short film that participants in prior studies have perceived as disturbing and that elicits intrusive thoughts. One week later, participants completed the Impact of Event Scale for the traumatic film, which measures the frequency and impact of intrusive thoughts about the target incident. Streb et al. found that individuals with better retrieval-suppression ability (whether measured behaviorally or electrophysiologically) reported significantly less distressing intrusions during the preceding week. Conversely, Catarino et al. (2015) found that participants with PTSD showed significantly less suppression-induced forgetting of unpleasant scenes and that suppression effects predicted participants’ symptom severity. Similar deficits in suppression-induced forgetting arise in people suffering from rumination and anxiety (e.g., Fawcett et al., 2015; Marzi, Regina, & Righi, 2014). Collectively, these findings suggest that in addition to reducing intentional explicit memory, retrieval suppression reduces involuntary retrievals.

## Suppression Reduces the Unintended Influence of Memory on Behavior

Even when people successfully control involuntary retrieval by purging unwanted memories from consciousness, suppressed memories could still influence behavior outside of awareness. To examine this possibility, several lines of research have employed indirect memory tests.

Hertel, Large, Stuck, and Levy (2012) used a free-association test to examine whether suppression arises on indirect tests. Participants first encoded cue-target word pairs and then participated in a TNT session. On a later free-association test, they were encouraged to report the first word that came to mind upon seeing a particular cue that they had encountered in the previous encoding session. Hertel et al. found that words that participants had previously suppressed during no-think trials were significantly less likely to be elicited in this free-association test. However, implicit memory tests that instead access participants’ memory for lower-level visual word form (e.g., a word’s orthography) have, in one study, not shown evidence of suppression (Angello, Storm, & Smith, 2015).

Subsequent research has shown that implicit suppression-induced-forgetting effects are not limited to conceptually oriented indirect tests but also impair perceptual repetition priming. In the first report of this, Kim and Yi (2013) asked participants to suppress retrieval of line drawings of visual objects. Later, participants performed a perceptual-identification task requiring them to identify briefly flashed images in visual noise. On such tests, people are usually better at identifying previously seen objects compared to novel items, a classic repetition-priming effect. Strikingly, across several experiments, Kim and Yi found that retrieval suppression significantly reduced repetition priming for no-think images. These findings indicate that retrieval suppression had counteracted the perceptual advantage normally enjoyed by repeated visual stimuli. Informatively, these implicit-suppression effects were abolished when test images were mirror-reversed upon repetition, suggesting that suppression directly inhibited perceptual representations (Kim & Yi, 2013). Consistent with this possibility, a study using photographs of real objects replicated reduced repetition priming and also observed reduced neural priming (i.e., repetition suppression) for the suppressed objects in visual object-perception regions (Gagnepain, Henson, & Anderson, 2014).

These demonstrations of reduced repetition priming have theoretical implications for the mechanisms underlying suppression-induced forgetting. For instance, putatively inhibitory effects observed on episodic cued-recall tests may instead reflect non-inhibitory mechanisms such as associative interference (e.g., Hertel & Calcaterra, 2005) or changes in context (Jonker, Seli, & MacLeod, 2015). Through these mechanisms, during the no-think task, the reminder cues become associated with alternative, distracting thoughts (associative interference) or with a new experimental context (context change); later, during the final cued-recall test, the reminder cues may now elicit either the alternative associations participants had formed (interference view) or the novel TNT-phase context associated with the reminder (context-change view), impairing memory for the original item, which is encountered only in the original study context. However, indirect tests such as perceptual identification do not require explicit recall but merely ask participants to perceive objects in visual noise; moreover, this task does not present the reminder cue from the TNT phase but only the visual object that is putatively inhibited, eliminating key preconditions of these mechanisms. Demonstrations of suppression-induced forgetting in this task, therefore, indicate that these alternative mechanisms are not sufficient to account for key phenomena and that item-specific inhibition is more likely. These findings echo work indicating that suppression-induced forgetting on episodic-memory tests is observed when suppressed items are tested with novel independent probes that circumvent interference (Anderson & Green, 2001; Wang, Cao, Zhu, Cai, & Wu, 2015; see Anderson & Hanslmayr, 2014, for a review).

**Fig. 2.**
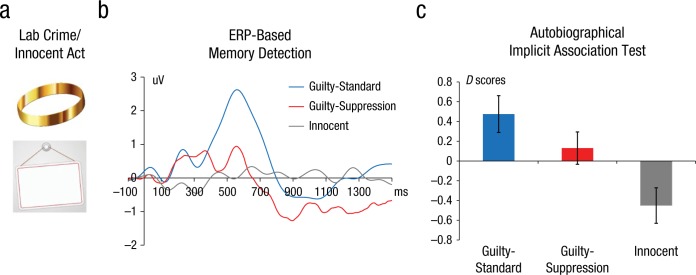
Results from Hu, Bergström, Bodenhausen, and Rosenfeld (2015) revealing the effects of suppressing unwanted autobiographical memories. “Guilty” participants enacted a lab crime in which they took a ring from a professor’s mailbox, whereas “innocent” participants wrote their initials on a board (a). Event-related potential (ERP) difference waves (ERP for crime-relevant stimulus—“ring”—minus the average ERP for crime-irrelevant stimuli—e.g., “wallet,” “bracelet”) revealed effects of retrieval suppression on autobiographical memory (b). A classic guilty-knowledge effect was evident among guilty participants without suppression instructions (guilty-standard group), as shown by enhanced retrieval-related ERP positivity during the 300- to 800-ms poststimulus window (for a recent review, see Rosenfeld, Hu, Labkovsky, Meixner, & Winograd, 2013). However, retrieval suppression largely attenuated this ERP positivity while enhancing the subsequent late posterior negativity (800–1,300 ms). Thus, individual guilty-suppression participants could be accurately detected when both ERP components were combined in a peak-to-peak manner. In an autobiographical Implicit Association Test (aIAT), compared to guilty-standard participants, guilty-suppression participants showed a significantly weaker implicit expression of their autobiographical memory (c). *D* scores reflected the strength of automatic activation of criminal memories and its unintentional influence on participants’ behavior (for rationales behind the aIAT and *D* scores, see Sartori, Agosta, Zogmaister, Ferrara, & Castiello, 2008). Error bars indicate 95% confidence intervals.

The foregoing findings indicate that retrieval suppression can reduce indirect effects of prior experience on cognition, at least for relatively simple materials. Recently, however, Hu, Bergström, Bodenhausen, and Rosenfeld (2015) extended this research by showing that suppression can reduce the unintentional influences of autobiographical memories with rich sensorimotor details. Participants engaged in a mock crime that involved taking a ring from a professor’s mailbox. They then completed an ERP memory-detection test wherein they were motivated to suppress retrieval of crime-relevant memories to avoid being detected. After the suppression phase, Hu et al. (2015) employed an autobiographical Implicit Association Test to indirectly measure the automatic activation of autobiographical memories (Hu, Rosenfeld, & Bodenhausen, 2012; Satori, Agosta, Zogmaister, Ferrara, & Castiello, 2008). Hu et al. found that the retrieval-related ERP positivity during the 300- to 800-ms poststimulus window was reduced during retrieval suppression (see also Bergström, Anderson, Buda, Simons, & Richardson-Klavehn, 2013) and, furthermore, that prior efforts to suppress retrieval had reduced the ability of the indirect test to detect automatic activation of crime-relevant memories in guilty participants (Fig. 2).

## Targeted Neocortical Inhibition as a Mechanism for Disrupting Unintended Retrieval

Evidence suggests that the need to countermand involuntary retrievals during retrieval suppression triggers inhibitory processes that down-regulate activity not only in the hippocampus but also in neocortical regions that support priming on indirect tests. The importance of intrusions was first demonstrated for the hippocampus. Using trial-by-trial intrusion reports, Levy and Anderson (2012) showed that retrieval suppression down-regulated hippocampal activity to a significantly greater extent during intrusion trials than during non-intrusions, and that only intrusion-related down-regulation predicted later suppression-induced forgetting. A later study found that negative coupling (an index of effective connectivity quantifying the inhibitory influence of one brain region on another) between the right dorsolateral prefrontal cortex and the hippocampus during early suppression trials predicted a greater decline in intrusions later in the TNT phase (Benoit et al., 2015), supporting the notion that top-down inhibitory control over memory-related regions (e.g., the hippocampus) gradually disrupts memories and renders them less likely to be involuntarily retrieved (Anderson, Bunce, & Barbas, 2016).

**Fig. 3.**
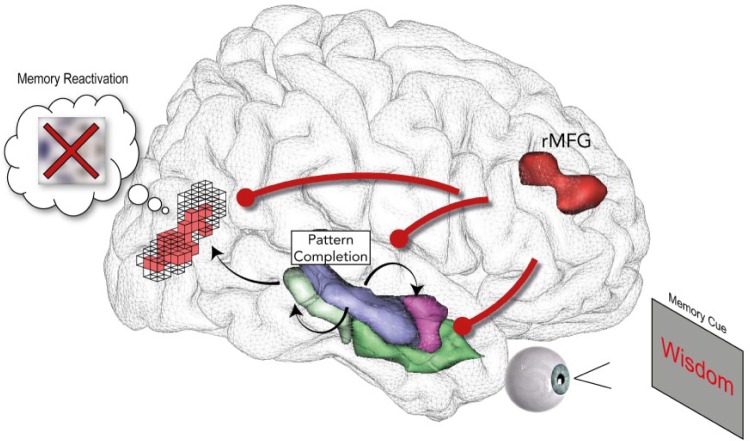
A schematic illustrating parallel, targeted inhibition of hippocampal, amygdala, and neocortical traces exerted by the prefrontal cortex during retrieval suppression. During retrieval suppression, sensory inputs from no-think reminders feed into the hippocampus (blue region), where they elicit pattern completion through reentrant connections to the amygdala (pink region), anterior and posterior parahippocampal gyrus (dark and light green regions, respectively), and visual cortex (black and white voxel grid). Completed patterns, symbolized here by red reactivated voxels in the visual cortex, reinstate neural activity that contributes to episodic experience (i.e., involuntary yet conscious intrusion) and interfere with goal-directed suppression. Such intrusions may trigger inhibitory control mediated by the right middle frontal gyrus (rMFG) to target both hippocampal and reactivated sites, gradually disrupting the corresponding neural/memory representations and impairing both intentional retrieval and unintentional memory expressions.

**Fig. 4.**
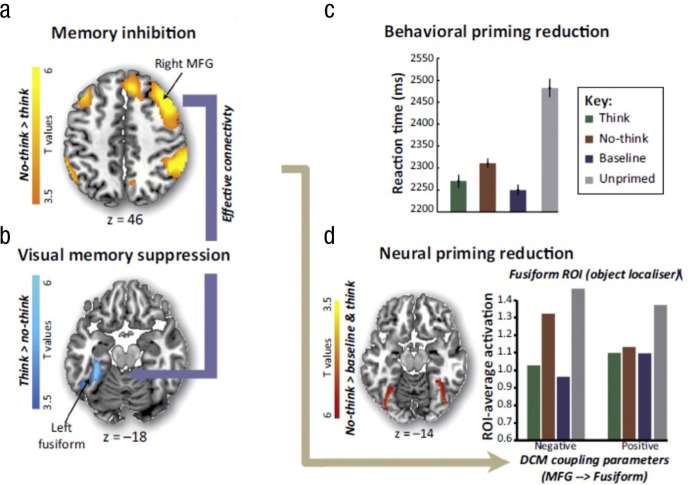
Results from Gagnepain, Henson, and Anderson (2014) showing that suppressing perceptual memories reduced subsequent perceptual priming on both behavioral and neural measures. Suppression recruited the right middle frontal gyrus (a) to down-regulate the left fusiform gyrus (b), as established via effective connectivity analyses. On a perceptual-identification test conducted after the think/no-think phase, reaction times revealed impaired behavioral priming effects for no-think trials compared to think and baseline trials (c). Results from fMRI scans during the final perceptual-identification task revealed impaired neural repetition-priming effects for no-think items (d; left), particularly when the right middle frontal gyrus had effectively down-regulated the left fusiform gyrus during the earlier think/no-think phase (d; right). Reprinted from “Neural Mechanisms of Motivated Forgetting,” by M. C. Anderson and S. Hanslmayr, 2014, *Trends in Cognitive Science, 18*, p. 288. Copyright 2014 by Elsevier.

Although hippocampal modulation is a key mechanism for controlling retrieval, control mechanisms also appear to target neocortical regions, particularly if neocortical traces are reactivated during intrusions. One broadly held view of retrieval is that perceptual reminders elicit pattern completion in the hippocampus, which, via reentrant connectivity with the neocortex, reinstates sensory neural patterns that contributed to the episodic experience (Danker & Anderson, 2010; McClelland, McNaughton, & O’Reilly, 1995). If intrusions also trigger such reinstatement, inhibitory control may also target neocortical traces to suppress retrieval (Fig. 3). This hypothesized targeting of neocortical representations by inhibitory control raises an important possibility: If neocortical traces support indirect expressions of memory on implicit tests, targeted neocortical inhibition may disrupt unintentional expressions of memory. Supporting this possibility, Gagnepain et al. (2014) found that when people suppressed episodic retrieval of visual object memories, the dorsolateral prefrontal cortex down-regulated activity not only in the hippocampus but also in visual object-perception regions in the fusiform cortex (see also Depue, Curran, & Banich, 2007). Importantly, a separate perceptual-identification test for the visual objects conducted after the TNT phase had ended revealed reduced neural priming for those objects that participants had suppressed from awareness. Critically, inhibitory modulation of the fusiform cortex (as measured by effective connectivity analyses) during the TNT phase predicted how much neural priming was disrupted on the later perceptual-identification test. These findings indicate that inhibitory control during retrieval suppression disrupted objects’ sensory representations, reducing the later ability of those sensory traces to indirectly enhance perception (see Fig. 4), consistent with the existence of item-specific inhibition.

Critically, the need to suppress reentrant activation of neocortical traces in this manner provides a general theoretical mechanism by which retrieval suppression could disrupt implicit memory across many content domains (Gagnepain et al., 2014). For instance, if reminders activate semantic representations associated with a memory item, suppression may disrupt conceptual priming (e.g., Hertel et al., 2012) via targeted activity-dependent inhibition of neocortical regions within the medial temporal lobe that support that type of priming (Anderson & Hanslmayr, 2014; Mayes, Montaldi, & Migo, 2007). Similarly, if reminders reactivate a memory’s emotional features, suppression may disrupt emotional traces via activity-dependent inhibition of amygdala activity (e.g., Depue et al., 2007). In the case of involuntary episodic remindings (conscious intrusions), reinstatement-dependent inhibition may jointly influence hippocampal and neocortical traces. Indeed, autobiographical retrieval engages the visual cortex and the hippocampus, possibly as a result of autobiographical memories’ rich sensory details (Cabeza & St. Jacques, 2007). Accordingly, suppressing autobiographical memories may target both the visual cortex and the hippocampus (see Noreen, O’Connor & MacLeod, 2016), reducing autobiographical memories’ unintentional influences. Thus, parallel, activity-dependent inhibition of hippocampal and neocortical traces may disrupt involuntary episodic retrievals and also impair implicit memory (Gagnepain et al., 2014).

## Conclusion

To free us from the influence of unwanted memories, retrieval suppression would ideally not only reduce their accessibility during intentional retrieval but also limit their unintended expressions. Here, we have reviewed recent evidence that suppression does, in fact, accomplish the latter function: It reduces memory intrusions and diminishes unwanted memories’ unintentional expressions in behavior. Reduced unintentional memories have been documented for a variety of content, ranging from verbal and simple perceptual to richly sensorimotor autobiographical memories. Neuroimaging research has, moreover, provided a key candidate mechanism for this function: When memory intrusions reactivate neocortical representations of to-be-suppressed memories via hippocampal pattern completion, both hippocampal and neocortical traces become targets for prefrontally mediated inhibitory-control processes. The top-down modulation of hippocampal and neocortical regions gradually disrupts the intruding traces, eventually modifying their unintended influences on later perception and cognition.

Many important questions await exploration. First, although retrieval suppression often succeeds and is beneficial, under some conditions, suppression appears to be counterproductive. For example, some people may fail to suppress retrieval effectively and suffer increased accessibility of unwanted traces as a result (Catarino et al., 2015), a problem of particular concern in psychiatric conditions characterized by deficits in inhibitory control. Moreover, even for healthy individuals, being asked to suppress a thought can increase its accessibility if the to-be-suppressed thought is part of the task instructions that need to be intermittently maintained in working memory, as in Wegner’s thought-suppression procedure (“Don’t think of a white bear”: Wegner, 1994; see Anderson & Huddleston, 2012, for a discussion). Clearly isolating how retrieval suppression differs from thought suppression and the conditions under which suppression succeeds or fails is a key priority. Second, although retrieval suppression reduces unintentional retrieval, results from related procedures, such as the list-method directed-forgetting paradigm, show that attempts to forget can impair intentional recall while leaving implicit memory intact (Bjork & Bjork, 2003). This difference suggests that some motivated-forgetting manipulations disrupt memory for individual items (retrieval suppression), whereas others instead may disrupt episodic context memory common to a set of items (directed forgetting; see Anderson, 2005, for a discussion), which may have important clinical implications. Third, a full understanding of how suppression affects memory requires an examination of its effects on reminders themselves. Interestingly, Hertel and Hayes (2015) recently showed that reminders for suppressed items captured more attention in a subsequent flanker task, likely because of repeated attention to these reminders during the TNT task.

More generally, however, the findings reviewed here suggest that it is useful for researchers and clinicians to reconsider the belief that suppression leaves unconscious expressions of memory intact. This pervasive belief might, in fact, arise precisely because psychopathological symptoms of interest to clinicians emerge in people who may have had preexisting deficits in memory-control capacity (Cole, Repovš, & Anticevic, 2014). In such individuals, suppression may indeed leave unintended expressions of memory intact, a possibility that can be tested experimentally. Ultimately, research on retrieval suppression holds the potential to develop a well-specified neurocognitive model concerning how people voluntarily control mnemonic awareness. Such a model could inform the development of interventions that would increase the integrity of the memory-control network, reduce intrusive thoughts, and improve mental health.

## References

[bibr1-0963721417689881] Anderson M. C. Hanslmayr S. (2014). (See References). An integrative review article on motivated memory control and its neural mechanisms.

[bibr2-0963721417689881] Gagnepain P. Henson R. N. Anderson M. C. (2014). (See References). Shows that suppressing perceptual memories impairs subsequent perceptual and neural priming effects and provides a neural model of the targeted cortical inhibition in retrieval suppression.

[bibr3-0963721417689881] Hertel P. T. Large D. Stuck E. D. Levy A. (2012). (See References). Shows that suppression reduces memory performance on a free-association test. 10.1080/09658211.2011.647036 22264096

[bibr4-0963721417689881] Hu X. Bergström Z. M. Bodenhausen G. V. Rosenfeld J. P. (2015). (See References). Shows that suppressing autobiographical memories weakens their subsequent unintended expression.

[bibr5-0963721417689881] Levy B. J. Anderson M. C. (2012). (See References). Demonstrates that retrieval suppression gradually reduces unwanted memories’ involuntary intrusions.

